# Plasmid-associated clonal expansion drives department-preference transmission of carbapenem-resistant *Klebsiella pneumoniae* in Xi’an, China: a genomic epidemiology study

**DOI:** 10.3389/fcimb.2025.1617222

**Published:** 2025-11-18

**Authors:** Kai Luo, Shuyan Liu, Jie Feng, Ruichao Li, Sirui Zhang, Rong Yu, Fang Li, Yali Li, Ke Wu, Juan Wang, Ting Xie, Jin’e Lei, Zhe Yin, Lei Han

**Affiliations:** 1 Department of Hepatobiliary Surgery, The First Affiliated Hospital of Xi’an Jiaotong University, Xi’an, China; 2 Department of Microbiology and Immunology, School of Basic Medical Sciences, Xi’an Jiaotong University Health Science Center, Xi’an, China; 3 Department of Otorhinolaryngology Head and Neck Surgery, The First Affiliated Hospital of Xi’an Jiaotong University, Xi’an, China; 4 State Key Laboratory of Medicinal Chemical Biology, Key Laboratory of Molecular Microbiology and Technology of the Ministry of Education, Department of Microbiology, College of Life Sciences, Nankai University, Tianjin, China; 5 Jiangsu Co-Innovation Center for Prevention and Control of Important Animal Infectious Diseases and Zoonoses, College of Veterinary Medicine, Yangzhou University, Yangzhou, China; 6 School of Public Health, Xi’an Jiaotong University, Xi’an, China; 7 Department of Laboratory Medicine, the First Affiliated Hospital of Xi’an Jiaotong University, Xi’an, China; 8 Key Laboratory of Bio-Resource and Eco-Environment of Ministry of Education, College of Life Sciences, Sichuan University, Chengdu, China; 9 College of Veterinary Medicine, Northwest A&F University, Yangling, China; 10 State Key Laboratory of Pathogen and Biosecurity, Academy of Military Medical Sciences, Beijing, China

**Keywords:** CRKP, clinical and genomic epidemiology, antibiotic resistance, virulence, plasmid

## Abstract

**Background:**

The drivers of expanding carbapenem-resistant *Klebsiella pneumoniae* (CRKP) infections in Northwest China remain poorly understood. This study aimed to investigate the clinical and molecular epidemiology of CRKP infections in Xi’an, China.

**Methods:**

We conducted a comprehensive analysis in a tertiary teaching hospital (September 2017–December 2019) by clinical data evaluation, antimicrobial susceptibility testing, and genome sequencing to characterize sequence types (STs), resistance and virulence genes, and plasmid profiles.

**Results:**

CRKP isolation rate increased from 3.6% to 17.2% during 2017-2019. Of 140 cases, 31.43% occurred in the Department of Hepatobiliary Surgery and 31 (22.14%) in the ICU, with a predominance among elderly male patients. Surgery and interventional therapy were performed in 50.7% and 25.7% of cases, respectively. Among 23 STs identified, ST11 (72.1%) was the most prevalent and exhibited high rate of multidrug resistance, commonly associated with narrow-spectrum β-lactamases-coding genes (highest in 110/140), *bla*_KPC-2_ (106/140), *rmtB* (85/140), *oqxAB* (110/140) and *fosA* (110/140). Virulence factors involved in secretion systems, iron uptake, and adhesion were identified. ST11 strains frequently carried IncFII, IncR and ColRNAI plasmids, while IncFIB-type was more relevant to ST147. Notably, strains harboring plasmid combinations IncFII_pHN7A8_:IncR:ColRNAI and IncFII_pHN7A8_:IncR:ColRNAI:IncFIB_(K)-1-kpn3_ were responsible for CRKP infections in the Department of Hepatobiliary Surgery and ICU, and exhibited higher resistance levels to carbapenems.

**Conclusions:**

Department-specific plasmid combinations considerably increased CRKP infection and multidrug resistance rates. Surgical and interventional therapies were vital factors contributing to CRKP infections, especially in elderly male patients.

## Introduction

*Klebsiella pneumoniae*, a Gram-negative bacterium belonging to *Enterobacterales*, is one of the major clinical and public health threats that can cause various types of infections including pneumonia, hepatic abscess, bloodstream and urinary tract infections (Zhu et al., 2021). The high mortality rate of *K. pneumoniae* infections is closely related to drug resistance and hypervirulence (Wang et al., 2020). Nowadays, drug-resistant *K. pneumoniae* isolates, particularly carbapenem-resistant *K. pneumoniae* (CRKP), have emerged as major threats in clinical settings (Martin and Bachman, 2018). CRKP spreads globally, with notable prevalence in North America, Europe, and Asia (Gonzalez-Ferrer et al., 2021). Specifically, during the COVID-19 pandemic, the number of carbapenem-resistant isolates and hypervirulent isolates increased in different continents (Liu et al., 2024). Similarly in China, there is a significant challenge in controlling the infections caused by CRKP.

Although phylogenetic and molecular epidemiological analyses, including popular strain typing, antimicrobial resistance mechanisms, and plasmid types, have been reported at the national and provincial levels (Hu et al., 2020; Liao et al., 2020), little is known about the clinical characteristics of CRKP infections and their correlation with molecular epidemiology. In addition, data on CRKP prevalence in Northwest China remain limited. Understanding the infectious characteristics and resistance mechanisms of CRKP is a critical step toward establishing therapeutic strategies.

Beyond drug resistance, enhanced virulence in *K. pneumoniae* strains also plays a crucial role in clinical infections. Generally speaking, classic *K. pneumoniae* (cKp) frequently causes hospital-acquired infections in weak patients, such as pneumonia and urinary tract infections (UTIs), due to its relatively mild pathogenesis. However, cKp can easily acquire multidrug resistance via mobile genetic elements (Gonzalez-Ferrer et al., 2021). In contrast, hypervirulent *K. pneumoniae* (hvKP) often causes community-acquired fulminant diseases, such as pyogenic liver abscess (PLA), endophthalmitis, and metastatic infection; it also exhibits a higher prevalence of causing pneumonia (Tang et al., 2025). Genes associated with high virulence are found on large virulence plasmids (Wang et al., 2020). Working together with the resistance plasmids, more dangerous strains expressing both carbapenem-resistant and hypervirulent phenotypes have emerged, referred to as CR-hvKP or hv-CRKP (Cejas et al., 2014; Gu et al., 2018; Jin et al., 2021). Thus, identifying virulence genes and their carriage elements is important for elucidating the evolutionary and epidemiological characteristics of clinical *K. pneumoniae* strains.

In the present study, we retrospectively collected and systematically analyzed 140 CRKP infection cases. Furthermore, based on the genome sequencing, we characterized various properties of these clinical strains and the factors involved in drug-resistance, virulence, and transmission, in order to uncover the epidemiology of CRKP in our hospital.

## Materials and methods

### Clinical strains and culture conditions

The CRKP isolation rates of China and Shaanxi Province were retrieved from the China Antimicrobial Resistance Surveillance System (CARSS, 2021), and that of our hospital (the First Affiliated Hospital of Xi’an Jiaotong University, XJFH) was reviewed from January 2017 to December 2019. Thereafter, a total of 140 CRKP strains were isolated from inpatients at XJFH between September 2017 and December 2019. All procedures conducted in this study received ethical approval from the Clinical Research Center and Ethics Committee of the First Affiliated Hospital of Xi’an Jiaotong University.

Clinical strains were cultivated in Luria-Bertani (LB) medium (Beijing Land Bridge Technology Co., Ltd., Beijing, China), and antimicrobial susceptibility testing was performed using cation-adjusted Muller-Hinton medium. Bacteria were incubated at 37°C, and subsequently stored at -80°C in LB medium containing 20% glycerol.

### Antimicrobial susceptibility testing

Antimicrobial susceptibility testing was performed using the VITEK 2 system (bioMérieux, Marcyl’étoile, France). Furthermore, the minimum inhibitory concentrations (MICs) of different antibiotics were confirmed using the broth microdilution method according to the guidelines of the Clinical and Laboratory Standards Institute (CLSI) (CLSI, 2024). The antibiotics included in this testing were piperacillin/tazobactam (TZP), ceftriaxone (CTX), ceftazidime (CAZ), ceftazidime/avibactam (CZA), cefepime (FEP), meropenem (MEM), imipenem (IPM), ertapenem (ETP), aztreonam (ATM), ciprofloxacin (CIP), amikacin (AMK), tigecycline (TGC), fosfomycin (FOS), cefoperazone/sulbactam (CSL), and polymyxin B (POL). Isolates resistant to agents from three or more antimicrobial classes were defined as multidrug-resistant (MDR) (Magiorakos et al., 2012).

### Genome sequencing and bioinformatic analysis

Genomic DNA from all CRKP strains was extracted using the TIANamp Bacteria DNA Kit (TIANGEN, Beijing, China) following the manufacturer’s instructions and stored at -80°C until use. DNA concentration was examined via a Qubit Fluorometer (Thermo Scientific, Waltham, MA, USA).

Genome sequencing was performed on an Illumina MiSeq platform (Illumina, San Diego, CA, USA), and sequence reads were assembled using SPAdes-3.13. Open reading frames (ORFs) were annotated through the Rapid Annotation using Subsystem Technology (RAST) pipeline (available at https://rast.nmpdr.org). Multilocus sequence typing (MLST) of our strains was analyzed using the BIGSdb-Pasteur database (https://bigsdb.pasteur.fr). Antibiotic resistance genes and virulence genes were identified using Resfinder (https://genepi.food.dtu.dk/resfinder) and Virulence Factor Database (VFDB, http://www.mgc.ac.cn/VFs), respectively, with a threshold of > 90% sequence identity. The evolutionary traits of the strains in correlation with carbapenemases were analyzed using MEGA 11. Plasmid replicons within the strains were identified using PlasmidFinder (https://cge.food.dtu.dk/services/PlasmidFinder) was utilized.

### Statistical analysis

Statistical analyses were performed using SPSS 27.0 (SPSS Inc, Chicago, IL. USA). The correlation between antibiotic resistance and sample sources was determined using the Chi-square test. The relationship between different plasmid combinations and the resistance levels to MEM, IPM and ETP was analyzed using the Kruskal-Wallis test. A *P* value < 0.05 was considered statistically significant for all tests. Graphs were generated using RStudio Version 4.3.3 (RStudio, Boston, MA, USA) with the ggplot2 package, GraphPad Prism Version 10.1.2 (GraphPad Software, San Diego, CA, USA), and Origin Pro 2021 (OriginLab Corporation, Northampton, MA, USA).

## Results

### Distribution and clinical impacts of CRKP infections

**Figure 1:**
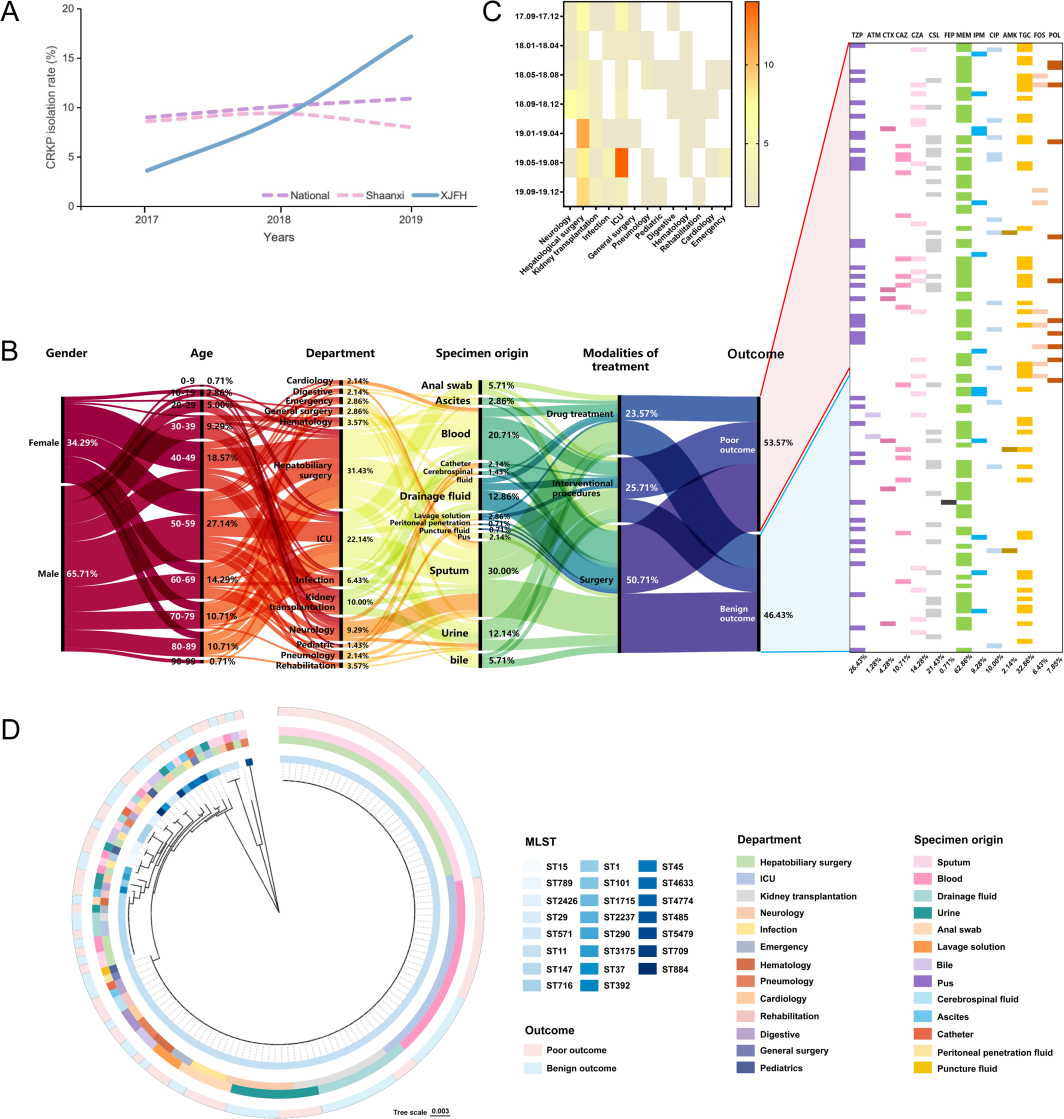
Clinical information and sample distribution of CRKP infection cases. **(A)** Trends in CRKP isolation rates at the national (China), provincial (Shaanxi Province), and hospital (First Affiliated Hospital of Xi’an Jiaotong University, XJFH) levels from January 2017 to December 2019. National and provincial data was obtained from the China Antimicrobial Resistance Surveillance System (CARSS, 2021). **(B)** General clinical information of infection cases and antibiotic usage during hospitalization. **(C)** Heatmap showing the three-month interval distribution of CRKP isolates from different departments. **(D)** Phylogenetic relationships of sequencing types (STs) with departments, specimens and outcomes. The concentric rings, from the innermost to the outermost, represent the STs of the isolates, the hospital departments where the isolates were obtained, the sample sources, and the clinical outcomes of the patients.

From 2017 to 2019, CRKP isolation rates in China and Shaanxi province remained relatively stable, fluctuating around 10%. Whereas, the isolation rate at our hospital (XJFH) increased notably, rising from 3.6% in 2017 to 17.2% in 2019 (**Figure 1A**).

To investigate the epidemiological drivers of this rapid increase, we collected 140 CRKP cases during September 2017 to December 2019. Samples were obtained from 13 departments, involving 92 male and 48 female patients. Two-thirds of the male patients were aged 50–89 years, while most female patients were 40–59 years old (**Figure 1B**). CRKP isolates were continuously isolated from the Department of Hepatobiliary Surgery (31.43%) and ICU (22.14%) (**Figures 1B, C**). Moreover, sputum (30.00%) and blood (20.71%) were the primary sample sources (**Figures 1B, D**). Of all patients, 50.70% underwent surgical treatment and 25.70% received interventional therapy. Additionally, the use of FOS and POL was significantly higher in patients with poor outcomes (10.67% vs. 1.54%, *P* = 0.0373; 13.33% vs. 1.54%, *P* = 0.0108) (**Figure 1B**).

### CRKP sequence types and their correlation with strain distribution

A total of 23 STs were identified, with ST11 being the dominant type (72.14%), followed by ST147 (7.14%). The remaining STs had frequencies ranging from 2.14% to 0.71% (**Figure 1D**).

ST11 was detected in 12 out of the 13 surveyed departments, excluding Pediatrics. Notably, the Department of Hepatobiliary Surgery exhibited the highest strain diversity (12 STs). No significant correlation between ST types of *K. pneumoniae* and patient prognosis was observed (**Figure 1D**).

### Antibiotic resistance patterns

**Figure 2:**
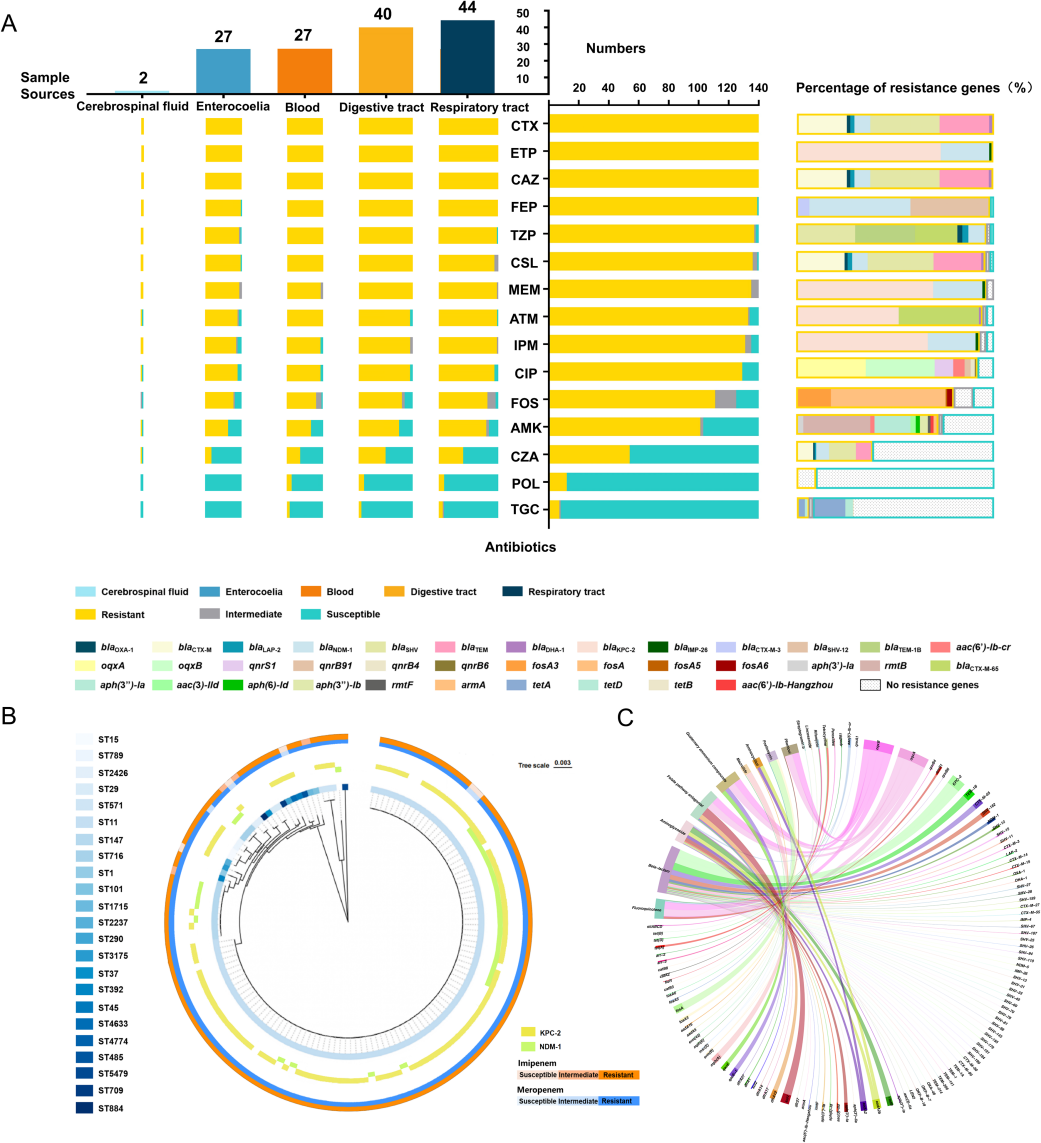
Relationship between antibiotic resistance patterns and resistance gene profiles, along with functional enrichment analysis of resistance genes. **(A)** General resistance patterns of 140 CRKP strains to 15 antibiotics (middle column), resistance patterns across five major sample sources (left column), and the proportion of resistance genes (right column). **(B)** Correlation of ST types with imipenem (IPM) and meropenem (MEM) resistance, along with the carbapenemase genes. The inner ring displays a cladogram of the 23 ST types. The middle ring shows the presence of two primary carbapenemase genes. The outer ring uses a two-color gradient to represent the resistance levels of 140 strains to MEM and IPM, with colors deepening from sensitive to resistant. **(C)** Genomic sequencing analysis of antibiotic resistance genes (ARGs) from 140 strains. The circular diagram depicts the functional enrichment of ARGs associated with 14 classes of antibiotics. CTX, cefotaxime; ETP, ertapenem; CAZ, ceftazidime; FEP, cefepime; TZP, piperacillin/tazobactam; CSL, cefoperazone/sulbactam; MEM, meropenem; ATM, aztreonam; IPM, imipenem; CIP, ciprofloxacin; FOS, fosfomycin; AMK, amikacin; CZA, ceftazidime/avibactam; POL, polymyxin; TGC, tigecycline.

Of the 140 isolates, 133 (95%) were MDR, and 7 (5%) were extensively drug-resistant (XDR). Strains displayed complete resistance to CTX, CAZ, and ETP, as well as relatively high resistance to FEP, TZP, CSL, MEM, ATM, IPM, and CIP, ranging downward from 99.29% to 92.14%. In contrast, high susceptibility was observed to POL (91.43%) and TGC (94.29%) (**Figure 2A**). A total of 36 distinct antimicrobial resistance combinations were identified (**Supplementary Table S1**).

Resistance patterns across different sample sources were consistent with the overall findings (**Figure 2A**). Strikingly, strains isolated from the respiratory tract exhibited the highest overall resistance rates, followed by those from blood and abdominal fluids.

### Identification of antibiotic resistance genes

A total of 106 ARGs were found in our strains (**Supplementary Table S2**). For the tested 15 antibiotics, which belong to six major classes, the predominant genes were associated with β-lactam resistance (**Supplementary Figure S1**). Specifically, *bla*_KPC-2_ (75.71%) and *bla*_NDM-1_ (27.14%) were the primary genes mediating carbapenem resistance (**Figures 2A, B**). Additionally, genes conferring resistance to fluoroquinolone (*oqxA* and *oqxB*), aminoglycosides (*rmtB*), and fosfomycin (*fosA*) were also relatively prevalent. Interestingly, *tetA* and *tetD* were found in both TGC-resistant and -sensitive strains, while no POL resistance genes were identified in any of the isolates (**Figure 2A**). Apart from them, ARGs responsible for the resistance to another nine classes of antibiotics were also discovered, among which, *sul1* (126/140), *mph*(A) (91/140), and *dfrA12* (70/140) were frequently detected (**Figure 2C**).

ST11 strains showed a strong correlation with ARG carriage, particularly with extended-spectrum β-lactamases (ESBLs) and carbapenemases genes (**Supplementary Figure S2**). Notably, the frequencies of *bla*_KPC-2_ and *bla*_NDM-1_ were significantly higher in ST11 strains than in other types (**Figure 2B**).

### Identification of virulence genes

**Figure 3:**
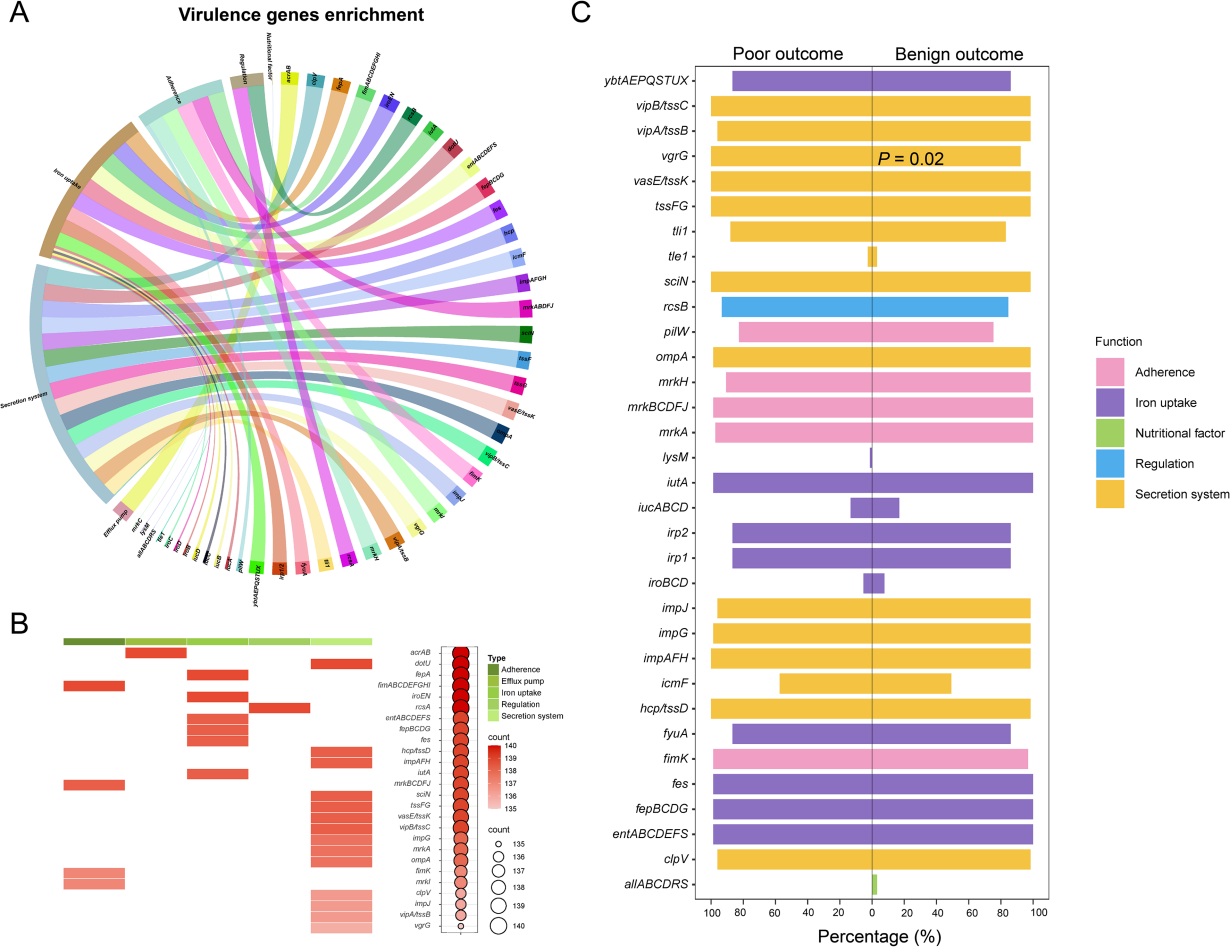
Genomic sequencing analysis of virulence genes from 140 CRKP strains. **(A)** Functional enrichment analysis of virulence genes. **(B)** Presence frequency of the top 52 virulence genes, along with their correlation with virulence functions. **(C)** Proportional distribution of virulence genes with different functions among patients with varying clinical outcomes.

From our isolates, 83 virulence genes were identified (**Figure 3A**). Among these, 16 genes were ubiquitously detected across all examined strains (from *acrAB* to *rcsA* in **Figure 3B**); while, 27 genes were present in 139 strains (from *entABCDEFS* to *vipB/tssC* in **Figure 3B**); furtherly, *ompA*, *impG* and *mrkA* were consistently identified in 138 strains. These most widely distributed genes were mainly involved in the functions of secretion, iron uptake, and adherence (**Figure 3A**).

The overall carriage rates of virulence genes did not differ significantly between patients with benign and poor outcomes (**Figure 3C**). However, when analyzing individual genes, *vgrG* exhibited a notably higher prevalence in patients with poor outcomes (*P* = 0.02). We further investigated the temporal trends in the number and type of virulence genes, and the results indicated that the detection rate of these genes was not associated with time (**Supplementary Figure S3**).

### Plasmids analysis

**Figure 4:**
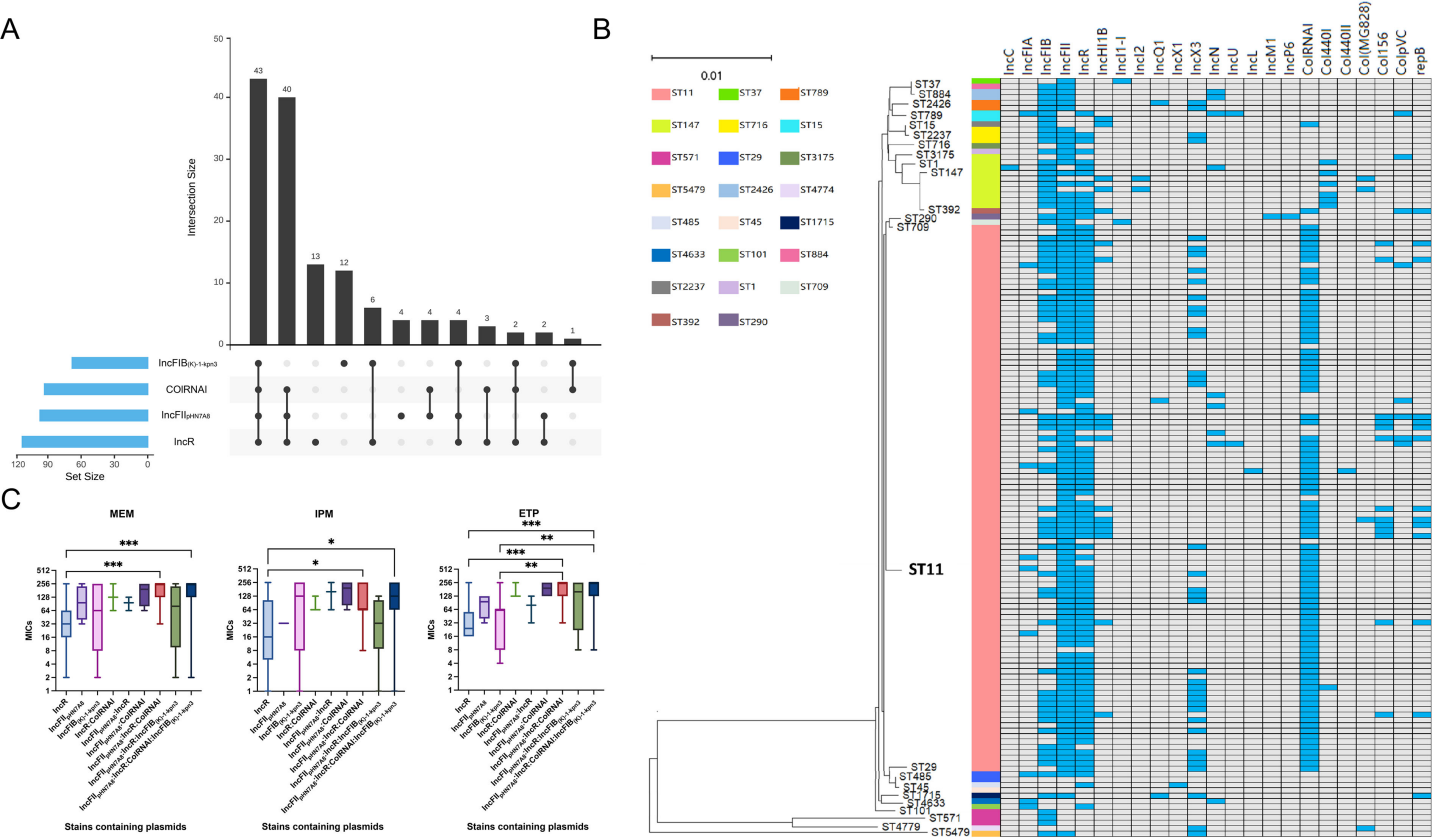
Analysis of four major plasmid types carried by CRKP. **(A)** Combinations and quantities of dominant plasmid types. **(B)** Association between plasmid types and ST types. Blue squares represent the presence of a plasmid type in a particular isolate, whereas grey squares indicate its absence. The neighbor-joining tree elucidates the phylogenetic relationships among the strains. **(C)** Correlation of different plasmid combinations with the resistance levels of CRKP strains to MEM, IPM, and ETP. MEM, meropenem; IPM, imipenem; ETP, ertapenem. *P* values were determined using the Kruskal–Wallis test. *, *P* < 0.05, **, *P* < 0.01, ***, *P* < 0.001.

A diverse range of plasmid groups was detected (**Supplementary Table S3**). IncFI plasmids were less existed, with IncFIB_(K)-1-kpn3_ (67/140) being the primary subtype. In contrast, IncFII was the most prevalent plasmid group (118/140), with IncFII_pHN7A8_ (97/140) as the dominant subtype. IncR (111/140) and ColRNAI (93/140) were also frequently observed. The distribution of the most dominant plasmid subtypes and their combinations is shown in **Figure 4A**.

**Figure 5:**
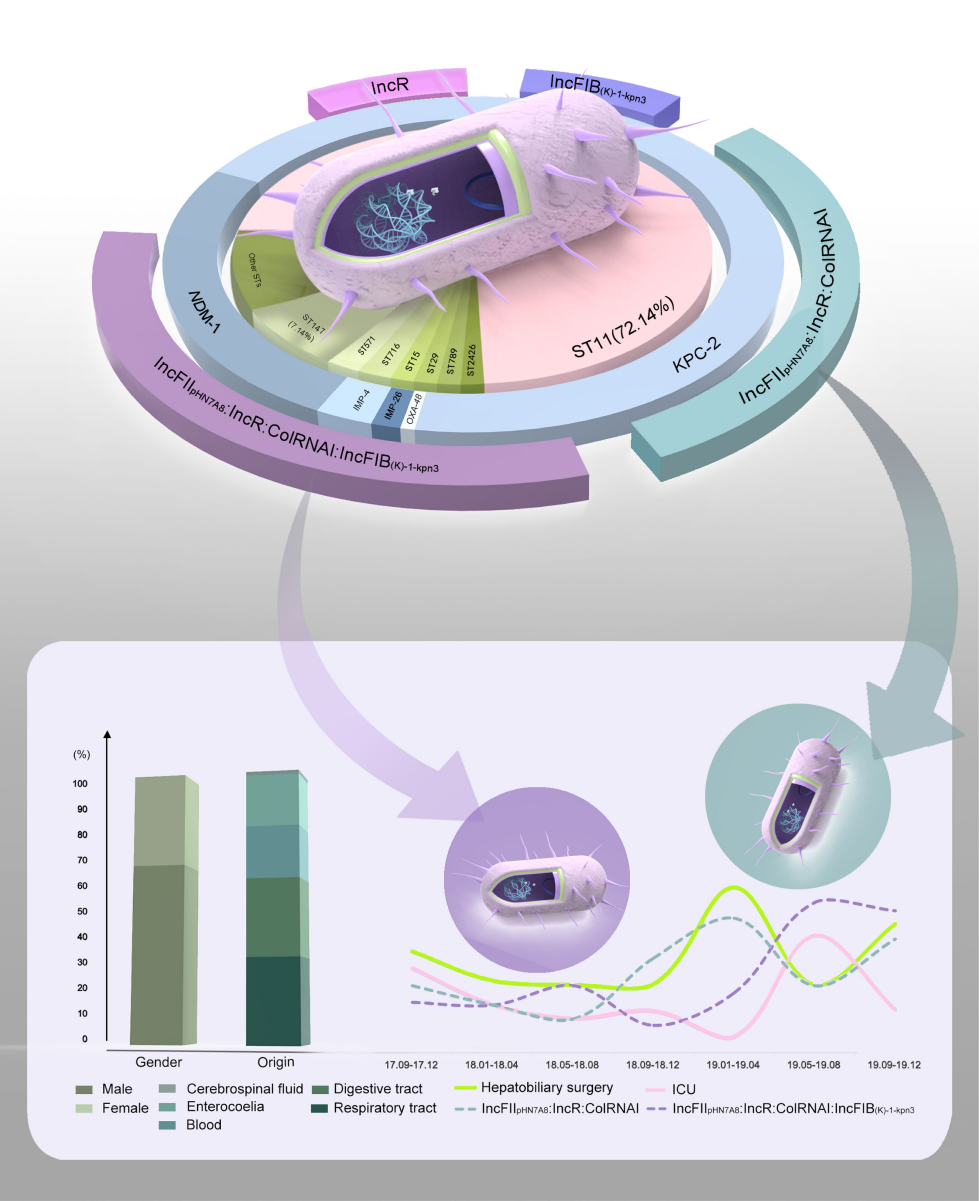
Phenotypic and genotypic characteristics of CRKP infections in our hospital. The pie chart illustrates the characteristics of 140 CRKP isolates, including ST types (inner ring), carbapenem resistance genes (middle ring), and primary plasmid combinations (outer ring). Gaps in the outer ring represent plasmid combinations with lower frequencies. The bar chart displays the age distribution of patients and the percentage of various sample sources. The line graph demonstrates a strong correlation between two plasmid combinations and CRKP infection prevalence. Specifically, IncFII_pHN7A8_:IncR:ColRNAI is associated with rates in the Department of Hepatobiliary Surgery, while IncFII_pHN7A8_:IncR:ColRNAI:IncFIB_(K)-1-kpn3_ correlates with prevalence in the ICU.

The vast majority of ST11 strains simultaneously carried IncFII, IncR and ColRNAI plasmids. While, IncFIB plasmids were more common in ST147 isolates (90%) than in ST11 isolates (47.52%) (**Figure 4B**). Intriguingly, strains carrying the plasmid combinations IncFII_pHN7A8_:IncR:ColRNAI and IncFII_pHN7A8_:IncR:ColRNAI: IncFIB_(K)-1-kpn3_ showed much higher MIC values for MEM, IPM, and ETP compared to strains carrying only IncR (MEM: *P* = 0.0001, *P* = 0.0007; IPM: *P* = 0.0457, *P* = 0.0488; ETP: *P* = 0.0001, *P* = 0.0001). An obvious difference was also noticed when comparing these combinations to strains carrying only IncFIB_(K)-1-kpn3_ for ETP (*P* = 0.0029 and *P* = 0.0037) (**Figure 4C**). Meanwhile, strains carrying IncFII_pHN7A8_:IncR:ColRNAI showed strong correlation with CRKP infections in the Department of Hepatobiliary Surgery, while those carrying IncFII_pHN7A8_:IncR: ColRNAI:IncFIB_(K)-1-kpn3_ were primarily associated with infections in the ICU (**Figure 5**).

## Discussion

CRKP is a life-threatening pathogen classified in the critical group of the WHO bacterial priority pathogen list (World Health Organization, 2024). Similar to the global rise in CRKP incidence, China also faces this public health challenge (Lin et al., 2024). However, the clinical characteristics of CRKP infections and its prevalence in Northwest China remain underdescribed.

During the investigation period, the isolation rate of CRKP in our hospital exhibited a notable increase, far exceeding the national and provincial levels in 2019. This upward trend was attributed to multiple contributing factors, including a general rise in overall hospital and ICU admissions, as well as an increase in the number of surgeries and invasive procedures. These observations are supported by a broader temporal context, which reveals both consistent and evolving trends in the global CRKP landscape. For instance, Shi et al. detected CRKP in 23.4% of KP-positive COVID-19 patients, identifying prolonged hospitalization and ICU admission as independent risk factors for CRKP acquisition (Shi et al., 2025). Furthermore, a surge in the number of carbapenem-resistant isolates was observed across multiple continents during the COVID-19 pandemic (Liu et al., 2024). Additional risk factors also played a role, including hepatobiliary disease, invasive medical procedures, renal failure, organ transplantation, and immunosuppression (Li et al., 2020; Chang et al., 2021). These factors may either disrupt the mucosal barrier at CRKP colonization site or directly facilitate pathogen access to sterile body sites, thereby enabling the establishment of infections (Chang et al., 2021; He et al., 2023). Indeed, more than three-fourths of our patients underwent surgeries and invasive manipulations. Whereas, different from the common observations that ICUs are the primary settings for CRKP infections (Liao et al., 2020), the Department of Hepatobiliary Surgery in our hospital exhibited the highest isolation rate. This was due to the increased surgical workload in the liver system and transplantation. In addition, male gender and older age are also two independent risk factors (Tofarides et al., 2023). Consistent with the findings of Tofarides et al (Tofarides et al., 2023), a higher proportion of male patients were infected with CRKP strains in this study. Moreover, elderly patients were more susceptible to CRKP infections, which aligns with the observations reported by Chang et al (Chang et al., 2021).

CRKP infections pose significant challenges in clinical practice, primarily due to their high resistance to multiple antibiotics (Vuotto et al., 2017). Notably, our strains showed even higher resistance rates to the tested antimicrobials, especially to cefotaxime, ceftazidime, and ertapenem. Furthermore, the soaring resistance rates to β-lactams, carbapenems, aminoglycoside, fluoroquinolone, and fosfomycin underscored a dire circumstance in which effective treatment options are severely limited. It is also noteworthy that the respiratory tract-derived *K. pneumoniae* displayed the highest resistance rates among all sources. Concurrently, bacteria isolated from both the digestive tract and blood showed complete resistance to seven antibiotics classified under the β-lactam and carbapenem classes, emphasizing the severity of antimicrobial resistance associated with these sites.

Numerous genetic elements were found responsible for the multiple resistance, particularly carbapenemase- and ESBL-encoding genes, *rmtB*, *oqxAB*, and *fosA*. KPC-2 was the most prevalent carbapenemase in our hospital, which has been shown to render strains with diverse susceptibility to imipenem and meropenem, but resistance to ertapenem (Ding et al., 2023). Notably, 23 isolates were identified as KPC-2-NDM-1-CRKP, which has been shown to exhibit high stability, fitness, and inter-host transmission capacity (Gao et al., 2020). In addition, a high frequency of CRKP strains carrying more than two ESBL-encoding genes was observed.

Given these challenges, there is an urgent need for effective therapeutic options, such as novel β-lactam/β-lactamase inhibitor (BL/BLI) combinations like ceftazidime/avibactam (CZA), imipenem/relebactam, and meropenem/vaborbactam. These agents exhibit strong activity against KPC-producing strains (Fratoni et al., 2024), which were predominant in our study. Indeed, our CRKP strains showed relatively high susceptibility to CZA, a finding consistent with the CHINET 2024 surveillance data (92.3%, https://www.chinets.com), supporting its role as a cornerstone treatment for CRKP infections. Additionally, *in vitro* studies have demonstrated the effectiveness of combination therapy with aztreonam and CZA against *Enterobacterales* expressing NDM and VIM enzymes (Fratoni et al., 2024). For CZA-resistant cases or when further alternatives are needed, imipenem-relebactam and meropenem-vaborbactam represent promising options, with CHINET 2024 surveillance reporting susceptibility rates of 96.0% and 94.0%, respectively. Beyond these new BL/BLIs, most of our strains remained susceptible to tigecycline and polymyxin, which are effective in treating complicated intra-abdominal infections and community-acquired pneumonia caused by MDR strains (Yang et al., 2023). Therefore, tigecycline was frequently used in our hospital. However, due to the potential side effects of polymyxin, it is reserved as a last-line treatment for critically ill patients. Whereas, attention need to be paid to TGC resistance due to the presence of *tet* genes (Du et al., 2018). Additionally, other mechanisms underlying polymyxin resistance should be considered, such as the disruption of the chromosomal *mgrB* gene by insertion sequences (Cannatelli et al., 2015). Taken together, these findings provide an evidence-based therapeutic framework for managing CRKP infections in clinical settings.

As the most prevalent CRKP clone of China (Liu et al., 2024), ST11 was not only strongly correlated with infection departments, sample sources and antibiotic resistance in our study, but also showed a close association with the plasmid types IncFII, IncR and ColRNAI. In contrast, IncFIB plasmids were more frequently related to ST147. Although the mechanism underlying plasmid preference in specific hosts remains unclear, previous studies suggested that successful high-risk CRKP clones are linked to specific narrow-host-range IncF plasmids, which may provide epidemiological advantages and stable environments for antimicrobial resistance genes over other clones (Mathers et al., 2015; Peirano et al., 2017). Indeed, strains carrying the plasmid combinations IncFII_pHN7A8_:IncR:ColRNAI and IncFII_pHN7A8_:IncR: ColRNAI:IncFIB_(K)-1-kpn3_ displayed higher resistance levels to carbapenems. More importantly, they were primarily responsible for CRKP dissemination in the Department of Hepatobiliary Surgery and ICU, respectively, with the ICU outbreak occurring later. This finding may be related to the clinical practice at our hospital, where patients who undergo hepatobiliary surgery are first monitored in the ICU, creating a potential opportunity for horizontal transmission of CRKP between these two units. In addition, patients in the Department of Hepatobiliary Surgery were primarily treated with third-generation cephalosporins for post-surgical bacterial prophylaxis, which may inadvertently select for CRKP and allow it to evade control. Furthermore, virulence factors involved in secretion, adherence, and iron acquisition were contributable to clinical infections. Intriguingly, *vgrG* was found positively associated with poorer outcomes in our study, verifying its critical role in CRKP pathogenicity (Li et al., 2024).

Overall, our research provides a detailed report on the clinical characteristics of local CRKP infections and identifies a specific transmission pattern mediated by chimera plasmids between the Department of Hepatobiliary Surgery and ICU. Enhanced monitoring of the molecular epidemiology of resistance and virulence profiles is pivotal for combating the escalating threat of CRKP and tailoring effective therapeutic and infection control strategies.

### Limitations

A limitation of this study, based on short-read sequencing data, is the inability to fully resolve complete plasmid structures or to definitively assign the physical location of resistance and virulence genes to specific plasmids. Future research employing long-read sequencing is warranted to fully elucidate the architecture of these hybrid plasmids and the precise genetic context of these critical genes.

## Data Availability

The genome sequences in this study have been deposited in the Genome Sequence Archive in National Genomics Data Center (NGDC), Beijing Institute of Genomics (BIG), Chinese Academy of Sciences (CAS) / China National Center for Bioinformation (CNCB) (GSA: CRA019634), and are publicly accessible at https://bigd.big.ac.cn/gsa/browse/CRA019634. The whole genome sequence data that support the findings in this study has been deposited in the Genome Warehouse in NGDC, BIG, CAS / CNCB (BioProject: PRJCA029853), and is publicly accessible at https://ngdc.cncb.ac.cn/gwh.

## Supplementary material

Supplementary Figure 1 Numbers of antibiotic resistance genes associated with five major classes of antibiotics.

Supplementary Figure 2 Composition of antibiotic resistance genes associated with 14 classes of antibiotics and their relationship with STs.

Supplementary Figure 3 Temporal analysis of virulence genes. Temporal changes in the relative abundance of all virulence genes, iron-uptake systems, nutrition factors, regulation, adhesins and secretion-system components among CRKP isolates collected during 2017–2019. Significance was assessed by two-tailed *t*-test. ns, *P* > 0.05.
