# Supplementary figures and images for: Plasmid-associated clonal expansion drives department-preference transmission of carbapenem-resistant Klebsiella pneumoniae in Xi’an, China: a genomic epidemiology study
Source: Front Cell Infect Microbiol. 2025 Nov 18;15:1617222. doi: 10.3389/fcimb.2025.1617222 (PMC12669119; doi:10.3389/fcimb.2025.1617222)

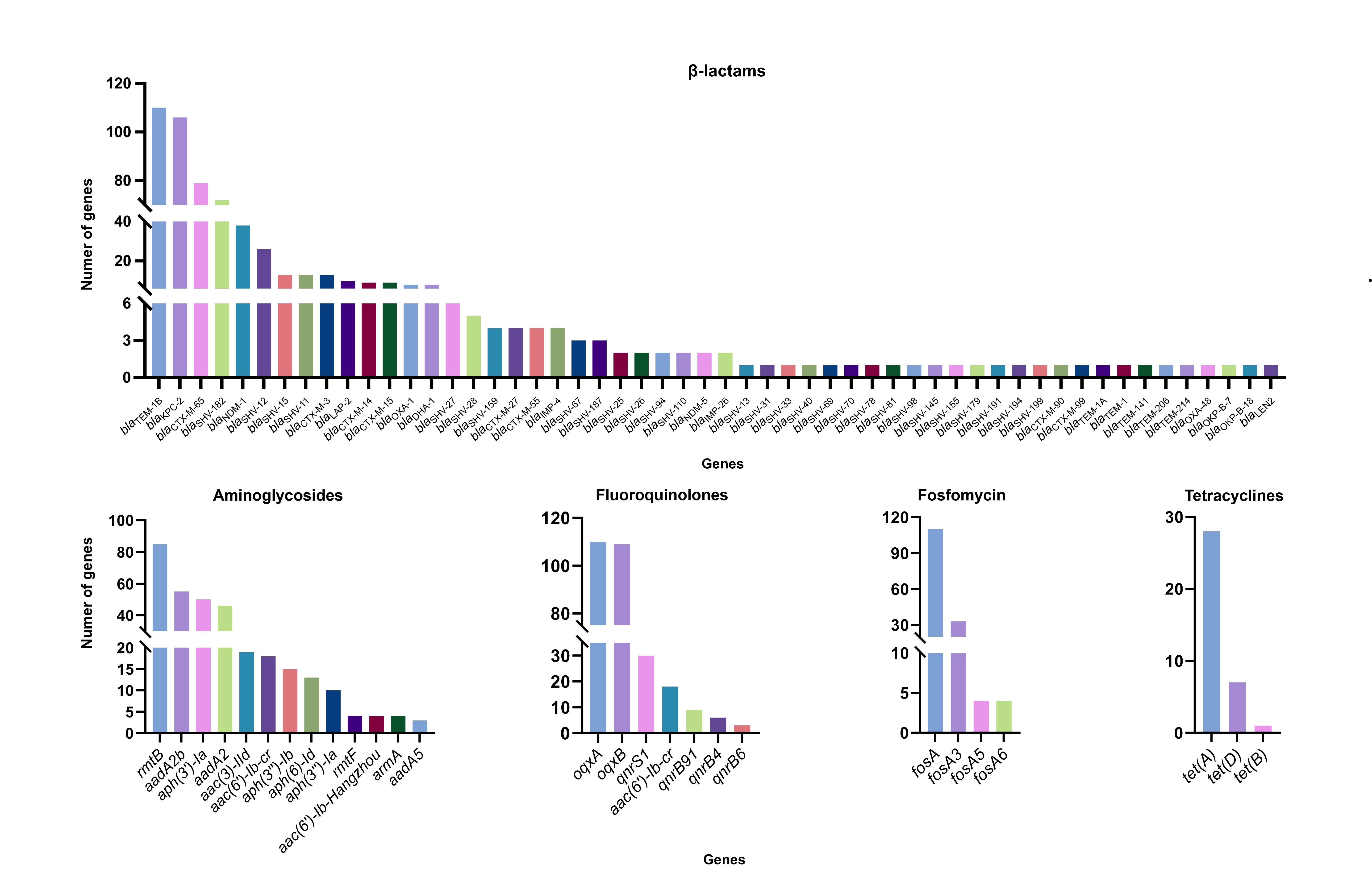

Supplement: Supplementary Figure 1 — Numbers of antibiotic resistance genes associated with five major classes of antibiotics. [file Image1.tif]

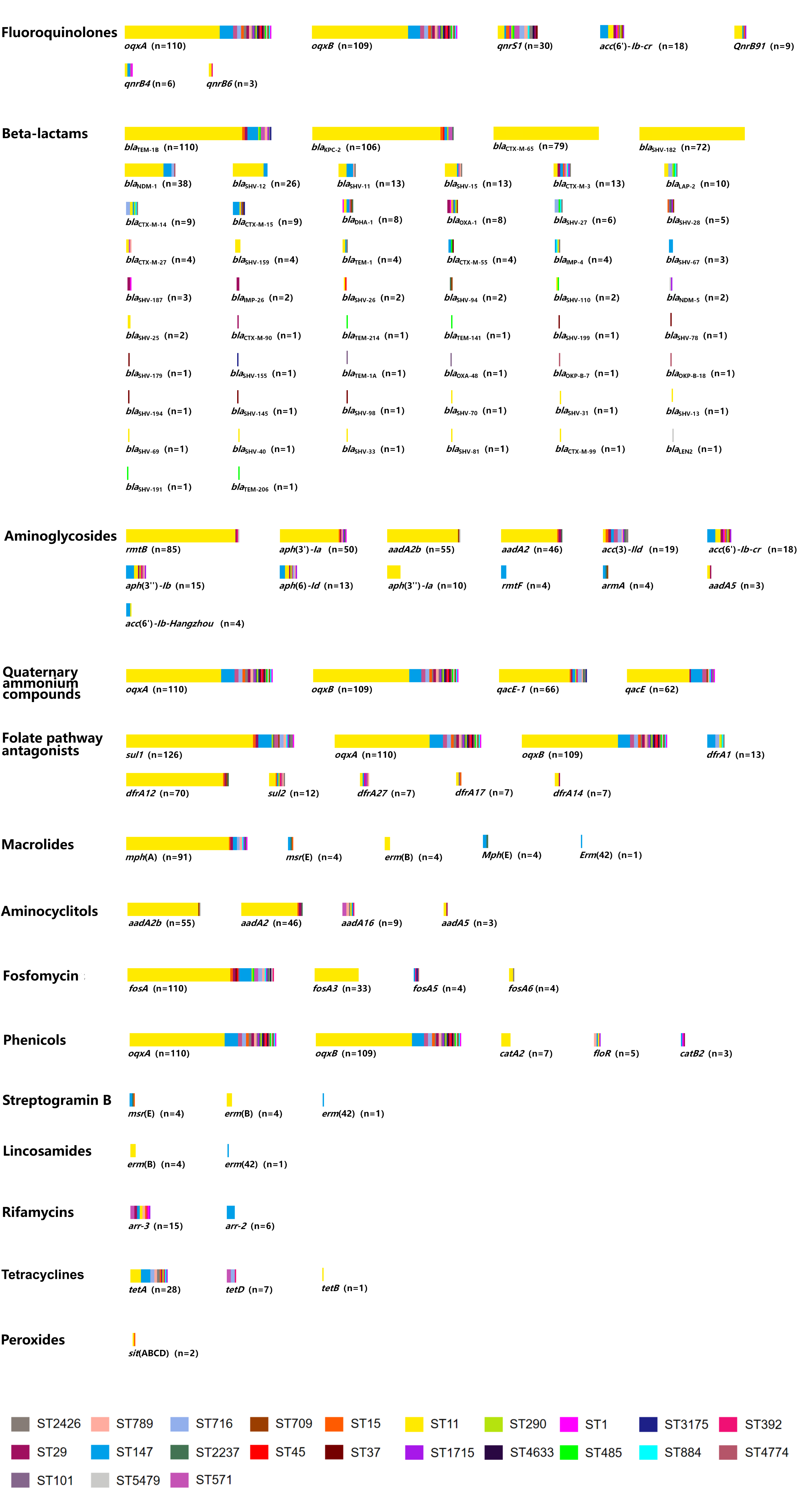

Supplement: Supplementary Figure 2 — Composition of antibiotic resistance genes associated with 14 classes of antibiotics and their relationship with STs. [file Image2.tif]

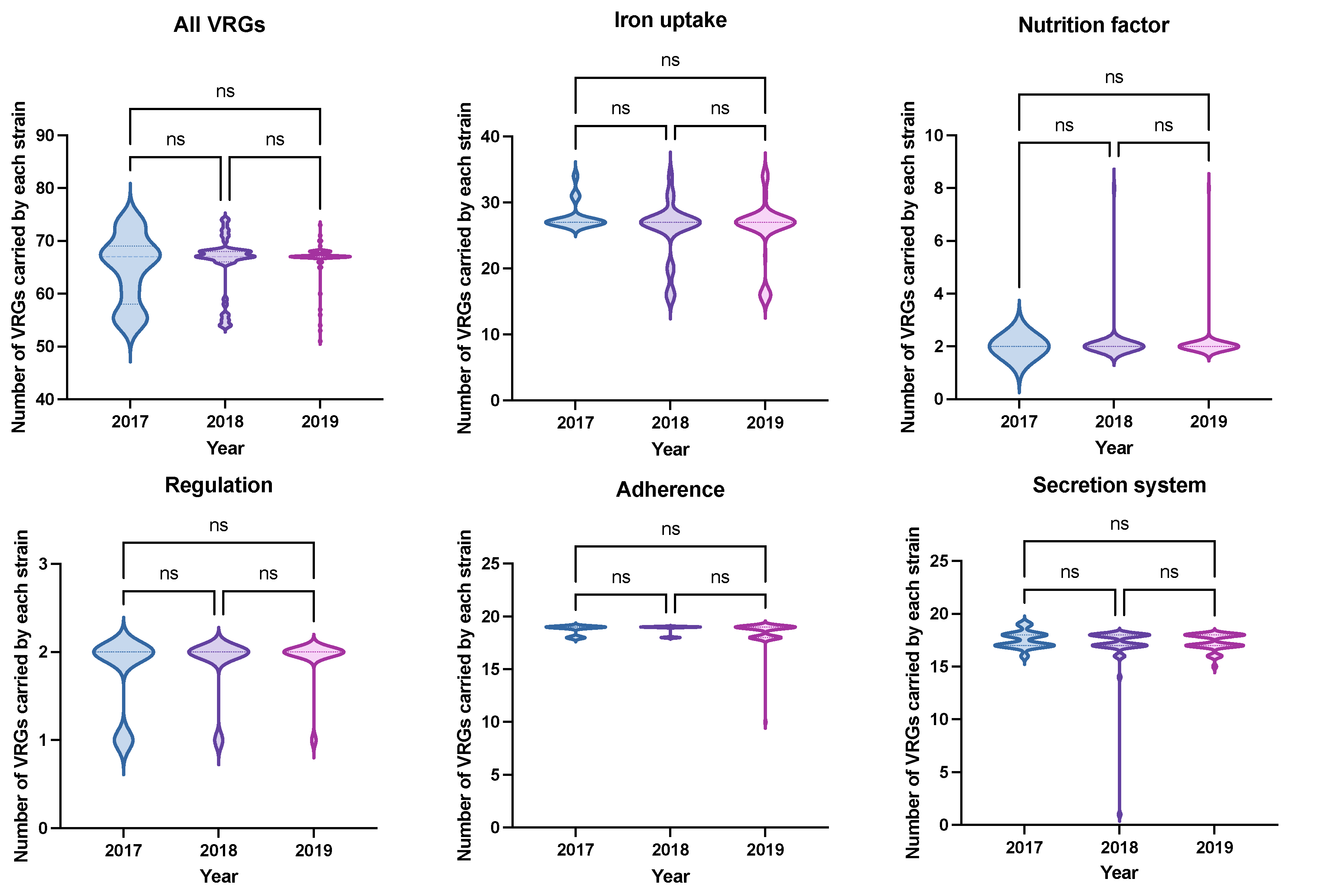

Supplement: Supplementary Figure 3 — Temporal analysis of virulence genes. Temporal changes in the relative abundance of all virulence genes, iron-uptake systems, nutrition factors, regulation, adhesins and secretion-system components among CRKP isolates collected during 2017–2019. Significance was assessed by two-tailed t-test. ns, P > 0.05. [file Image3.tif]
