# Supplementary material for: Plasmid-associated clonal expansion drives department-preference transmission of carbapenem-resistant Klebsiella pneumoniae in Xi’an, China: a genomic epidemiology study
Source: Front Cell Infect Microbiol. 2025 Nov 18;15:1617222. doi: 10.3389/fcimb.2025.1617222 (PMC12669119; doi:10.3389/fcimb.2025.1617222)
Supplement: Supplementary file 4 [file Table1.docx]

Table S1 Relationship of STs with antibiotic resistance profiles.

| Sequence type (ST) | Number | Antibiotic resistance profile | Number | Proportion |
| --- | --- | --- | --- | --- |
| 11 | 101 | CZA, AMK, FEP, CAZ, TZP, CSL, ETP, IPM, MEM, CIP, ATM, CTX, FOS | 18 | 12.86% |
|  |  | CZA, AMK, FEP, CAZ, TZP, CSL, ETP, IPM, MEM, CIP, ATM, CTX | 3 | 2.14% |
|  |  | CZA, AMK, FEP, CAZ, TZP, CSL, ETP, IPM, MEM, CIP, POL, FOS, ATM, CTX | 8 | 5.71% |
|  |  | CZA, AMK, FEP, CAZ, TZP, CSL, ETP, IPM, MEM, CIP, POL, ATM, CTX | 1 | 0.71% |
|  |  | CZA, AMK, FEP, CAZ, TZP, ETP, IPM, MEM, CIP, FOS, ATM, CTX | 1 | 0.71% |
|  |  | CZA, AMK, FEP, CAZ, TGC, TZP, CSL, ETP, IPM, MEM, CIP, FOS, ATM, CTX | 2 | 1.43% |
|  |  | CZA, FEP, CAZ, TZP, CSL, ETP, IPM, MEM, CIP, FOS, ATM, CTX | 2 | 1.43% |
|  |  | CZA, FEP, CAZ, TZP, CSL, ETP, IPM, MEM, FOS, ATM, CTX | 2 | 1.43% |
|  |  | CZA, FEP, CAZ, TZP, CSL, ETP, IPM, MEM, ATM, CTX | 1 | 0.71% |
|  |  | CZA, FEP, CAZ, CSL, ETP, IPM, MEM, CTX | 1 | 0.71% |
|  |  | AMK, FEP, CAZ, TZP, CSL, ETP, IPM, MEM, CIP, FOS, ATM, CTX | 41 | 29.29% |
|  |  | AMK, FEP, CAZ, TZP, CSL, ETP, IPM, MEM, CIP, ATM, CTX | 9 | 6.43% |
|  |  | AMK, FEP, CAZ, TZP, CSL, ETP, IPM, MEM, CIP, POL, FOS, ATM, CTX | 2 | 1.43% |
|  |  | AMK, FEP, CAZ, TZP, CSL, ETP, CIP, FOS, ATM, CTX | 2 | 1.43% |
|  |  | AMK, FEP, CAZ, TGC, TZP, CSL, ETP, IPM, MEM, CIP, FOS, ATM, CTX | 1 | 0.71% |
|  |  | FEP, CAZ, TZP, CSL, ETP, IPM, MEM, CIP, FOS, ATM, CTX | 5 | 3.57% |
|  |  | FEP, CAZ, TZP, CSL, ETP, IPM, MEM, CIP, ATM, CTX | 1 | 0.71% |
|  |  | FEP, CAZ, TZP, ETP, IPM, MEM, FOS, ATM, CTX | 1 | 0.71% |
| 147 | 10 | CZA, AMK, FEP, CAZ, TZP, CSL, ETP, IPM, MEM, CIP, FOS, ATM, CTX | 2 | 1.43% |
|  |  | CZA, AMK, FEP, CAZ, TZP, CSL, ETP, IPM, MEM, CIP, ATM, CTX | 1 | 0.71% |
|  |  | CZA, FEP, CAZ, TZP, CSL, ETP, IPM, MEM, CIP, FOS, ATM, CTX | 1 | 0.71% |
|  |  | CZA, FEP, CAZ, CSL, ETP, IPM, MEM, CIP, CTX | 1 | 0.71% |
|  |  | CZA, FEP, CAZ, TZP, CSL, ETP, IPM, MEM, CIP, ATM, CTX | 1 | 0.71% |
|  |  | FEP, CAZ, TZP, CSL, ETP, MEM, CIP, FOS, ATM, CTX | 1 | 0.71% |
|  |  | AMK, FEP, CAZ, TGC, TZP, CSL, ETP, IPM, MEM, CIP, FOS, ATM, CTX | 1 | 0.71% |
|  |  | AMK, FEP, CAZ, TZP, CSL, ETP, IPM, MEM, CIP, FOS, ATM, CTX | 1 | 0.71% |
|  |  | AMK, FEP, CAZ, TZP, ETP, IPM, MEM, CIP, FOS, ATM, CTX | 1 | 0.71% |
| 571 | 3 | CZA, FEP, CAZ, TZP, CSL, ETP, IPM, MEM, CIP, FOS, ATM, CTX | 2 | 1.43% |
|  |  | FEP, CAZ, TGC, TZP, CSL, ETP, IPM, CIP, ATM, CTX | 1 | 0.71% |
| 716 | 3 | AMK, FEP, CAZ, TGC, TZP, CSL, ETP, IPM, MEM, CIP, FOS, ATM, CTX | 1 | 0.71% |
|  |  | FEP, CAZ, TZP, CSL, ETP, IPM, MEM, CIP, FOS, ATM, CTX | 2 | 1.43% |
| 15 | 2 | AMK, FEP, CAZ, TZP, CSL, ETP, IPM, MEM, CIP, FOS, ATM, CTX | 1 | 0.71% |
|  |  | AMK, CAZ, TZP, CSL, ETP, IPM, MEM, CIP, FOS, ATM, CTX | 1 | 0.71% |
| 29 | 2 | CZA, FEP, CAZ, TGC, TZP, CSL, ETP, IPM, MEM, CIP, CTX | 1 | 0.71% |
|  |  | CZA, FEP, CAZ, TZP, CSL, ETP, IPM, MEM, CTX | 1 | 0.71% |
| 789 | 2 | FEP, CAZ, TZP, CSL, ETP, IPM, MEM, CIP, FOS, ATM, CTX | 1 | 0.71% |
|  |  | AMK, FEP, CAZ, TZP, CSL, ETP, IPM, MEM, CIP, FOS, ATM, CTX | 1 | 0.71% |
| 2426 | 2 | CZA, FEP, CAZ, CSL, ETP, MEM, CTX | 1 | 0.71% |
|  |  | FEP, CAZ, TZP, CSL, ETP, CIP, FOS, ATM, CTX | 1 | 0.71% |
| 37 | 2 | FEP, CAZ, TZP, CSL, ETP, IPM, MEM, ATM, CTX | 1 | 0.71% |
|  |  | FEP, CAZ, TZP, CSL, ETP, IPM, MEM, CIP, FOS, ATM, CTX | 1 | 0.71% |
| 24 | 2 | FEP, CAZ, TZP, CSL, CIP, ATM, CTX | 1 | 0.71% |
|  |  | FEP, CAZ, TZP, CSL, MEM, CIP, FOS, ATM, CTX | 1 | 0.71% |
| 80 | 2 | FEP, CAZ, TZP, CSL, MEM, CIP, FOS, ATM, CTX | 1 | 0.71% |
|  |  | FEP, CAZ, TZP, CSL, MEM, FOS, ATM, CTX | 1 | 0.71% |
| 97 | 2 | CAZ, TZP, ETP, IPM, MEM, CIP, FOS, ATM, CTX | 1 | 0.71% |
|  |  | AMK, FEP, CAZ, TZP, CSL, ETP, IPM, MEM, CIP, POL, FOS, ATM, CTX | 1 | 0.71% |
| 142 | 2 | AMK, FEP, CAZ, TZP, CSL, ETP, IPM, MEM, CIP, FOS, ATM, CTX | 2 | 1.43% |
| 51 | 2 | AMK, FEP, CAZ, TZP, CSL, ETP, IPM, MEM, CIP, ATM, CTX | 1 | 0.71% |
|  |  | CZA, FEP, CAZ, TZP, CSL, ETP, MEM, CTX | 1 | 0.71% |
| 92 | 2 | CZA, FEP, CAZ, TZP, CSL, ETP, MEM, CTX | 1 | 0.71% |
|  |  | CZA, FEP, CAZ, TZP, CSL, ETP, MEM, CIP, ATM, CTX | 1 | 0.71% |
| 106 | 1 | CZA, FEP, CAZ, TZP, CSL, ETP, MEM, CIP, FOS, ATM, CTX | 1 | 0.71% |

CZA, ceftazidime-avibactam; AMK, amikacin; FEP, cefepime; CAZ, ceftazidime; TZP, piperacillin-tazobactam; CSL, cefoperazone/sulbactam; ETP, ertapenem; IPM, imipenem; MEM, meropenem; CIP, ciprofloxacin; CTX, ceftriaxone; ATM, aztreonam; TGC, tigecycline; FOS, fosfomycin; and POL, polymyxin B.

Table S2 Numbers of detected antibiotic resistance genes.

| Class of antibiotics | Antibiotic resistance genes | Number of strains |
| --- | --- | --- |
| Peroxides | *sitABCD* | 12 |
| Tetracyclines | *tet(A)*  *tet(B)*  *tet(D)* | 28  1  7 |
| Rifamycin | *arr-2*  *arr-3* | 6  15 |
| Lincosamidea | *erm(B)*  *erm(42)* | 4  1 |
| Streptogramin B | *erm(B)*  *erm(42)*  *msr(E)* | 4  1  4 |
| Phenicols | *oqxA*  *oqxB*  *floR*  *catA2*  *catB3*  *catB8* | 110  109  5  7  3  1 |
| Fosfomycin | *fosA*  *fosA3*  *fosA5*  *fosA6* | 110  33  4  4 |
| Aminocyclitols | *aadA2*  *aadA2b*  *aadA5*  *aadA16* | 46  55  3  9 |
| Macrolides | *erm(B)*  *erm(42)*  *msr(E)*  *mph(A)*  *mph(E)* | 4  1  4  91  4 |
| Quaternary ammonium compounds | *oqxA*  *oqxB*  *qacE*  *qacE-1* | 110  109  62  66 |
| Folate pathway antagonists | *dfr27*  *dfrA1*  *dfrA12*  *dfrA14*  *dfrA17*  *dfrA27*  *oqxA*  *oqxB*  *sul1*  *sul2* | 1  13  70  4  4  7  110  109  126  12 |
| Aminoglycosides | *aac(3)-Iia*  *aac(3)-IId*  *aac(6')-Ib-cr*  *aac(6')-Ib-Hangzhou*  *aadA2*  *aadA2b*  *aph(3')-Ia*  *aph(3'')-Ia*  *aph(3')-Iia*  *aph(3'')-Ib*  *aph(6)-Id*  *armA*  *rmtB*  *rmtF* | 1  19  20  4  46  55  50  10  1  15  13  4  85  4 |
| Narrow-spectrum β-lactamases  ESBLs  Carbapenemases  Narrow-spectrum β-lactamases  Carbapenemases  Plasmid-mediated AmpC β-lactamase | *bla*_SHV-11_  *bla*_SHV-12_  *bla*_SHV-13_  *bla*_SHV-15_  *bla*_SHV-25_  *bla*_SHV-26_  *bla*_SHV-27_  *bla*_SHV-28_  *bla*_SHV-31_  *bla*_SHV-33_  *bla*_SHV-40_  *bla*_SHV-67_  *bla*_SHV-69_  *bla*_SHV-70_  *bla*_SHV-78_  *bla*_SHV-81_  *bla*_SHV-94_  *bla*_SHV-98_  *bla*_SHV-110_  *bla*_SHV-145_  *bla*_SHV-155_  *bla*_SHV-159_  *bla*_SHV-179_  *bla*_SHV-182_  *bla*_SHV-187_  *bla*_SHV-191_  *bla*_SHV-194_  *bla*_SHV-199_  *bla*_CTX-M-3_  *bla*_CTX-M-14_  *bla*_CTX-M-15_  *bla*_CTX-M-27_  *bla*_CTX-M-55_  *bla*_CTX-M-65_  *bla*_CTX-M-90_  *bla*_CTX-M-99_  *bla*_TEM-1A_  *bla*_TEM-1B_  *bla*_TEM-1_  *bla*_TEM-141_  *bla*_TEM-206_  *bla*_TEM-214_  *bla*_NDM-1_  *bla*_NDM-5_  *bla*_OXA-1_  *bla*_OXA-48_  *bla*_IMP-4_  *bla*_IMP-26_  *bla*_OKP-B-7_  *bla*_OKP-B-18_  *bla*_LEN2_  *bla*_KPC-2_  *bla*_LAP-2_  *bla*_DHA-1_ | 13  26  1  13  2  2  6  5  1  1  1  3  1  1  1  1  2  1  2  1  1  4  1  72  3  1  1  1  13  9  9  4  4  79  1  1  1  110  1  1  1  1  38  2  8  1  4  2  1  1  1  106  10  8 |
| Fluoroquinolones | *aac(6')-Ib-cr*  *oqxA*  *oqxB*  *qnrA1*  *qnrB4*  *qnrB6*  *qnrB91*  *qnrS1* | 20  110  109  1  6  3  9  30 |

Table S3 Plasmid types carried by CRKP strains in our study.

| Strain No. | Plasmid types |
| --- | --- |
| 1 | IncFIB_AP001918_, IncFII_pHN7A8_, IncR, ColRNAI |
| 2 | IncFII_pHN7A8_, IncFII_p14_, IncR, ColRNAI |
| 3 | IncFIB_(K)-1-kpn3_, IncFII_pHN7A8_, IncR, IncX3, ColRNAI |
| 4 | IncFIA, IncFIB_(K)-1-kpn3_, IncFII_pHN7A8_, IncR |
| 5 | IncFIB_(K)-1-kpn3_, IncFII_pHN7A8_, IncR, ColRNAI, Col156, repB |
| 6 | IncFII_pHN7A8_, IncFII_pKP91_, IncR, IncX3, ColRNAI |
| 7 | IncFIB_(K)-1-kpn3_, IncFII_pHN7A8_, IncR, IncX3 |
| 8 | IncFIB_(K)-1-kpn3_, IncFII_pHN7A8_, IncR, ColRNAI, Col156, repB |
| 9 | IncFIA, IncFII_pHN7A8_, IncFII_pKP91_, IncFII_pKPX1_, ColpVC |
| 10 | IncFIB_(K)-1-kpn3_, IncFIB_AP001918_, IncFII_pKP91_, IncR, IncX3, ColRNAI |
| 11 | IncFII_pHN7A8_, IncFII_pKP91_, IncR, ColRNAI |
| 12 | IncFIB_(K)-1-kpn3_, IncFII_pHN7A8_, IncFII_pKP91_, IncR, IncX3, ColRNAI |
| 13 | IncFIB_(K)-1-kpn3_, IncFII_pHN7A8_, IncR |
| 14 | IncFII_pHN7A8_, IncR, ColRNAI |
| 15 | IncFIB_(K)-1-kpn3_, IncFII_pHN7A8_, IncR, IncX3, ColRNAI |
| 16 | IncFIB_(K)-1-kpn3_, IncFII_pHN7A8_, IncR, ColRNAI |
| 17 | IncFII_pKP91_, IncFII_pCTU2_, IncR, IncI2 |
| 18 | IncFIB_(K)-1-kpn3_, IncFII_pHN7A8_, IncFII_pKP91_, IncR, IncX3, ColRNAI |
| 19 | IncFIB_(K)-1-kpn3_, IncFII_pHN7A8_, IncR, IncX3, ColRNAI |
| 20 | IncFIB_(K)-1-kpn3_, IncFII_pHN7A8_, IncR, ColRNAI |
| 21 | IncFII_pHN7A8_, IncFII_pKP91_, IncR, ColRNAI |
| 22 | IncFII_pHN7A8_, IncFII_pKP91_, IncR, IncX3, ColRNAI |
| 23 | IncFIB_(K)-1-kpn3_, IncFII_pHN7A8_, IncFII_pKP91_, IncR, IncX3, ColRNAI |
| 24 | IncFIB_(K)-1-kpn3_, IncFII_pHN7A8_, IncR, ColRNAI |
| 25 | IncFIB_(K)-1-kpn3_, IncFII_pHN7A8_, IncR, ColRNAI |
| 26 | IncFII_pHN7A8_, IncFII_pKP91_, IncR, ColRNAI |
| 27 | IncFII_pHN7A8_, IncR, ColRNAI |
| 28 | IncFIB_(K)-1-kpn3_, IncFII_pHN7A8_, IncR, ColRNAI |
| 29 | IncFII_pHN7A8_, IncR, ColRNAI |
| 30 | IncFII_pHN7A8_, IncR, ColRNAI |
| 31 | IncFIB_(K)-1-kpn3_, IncFIB_AP001918_, IncFII_pHN7A8_, IncR, IncX3, ColRNAI |
| 32 | IncFIB_(K)-1-kpn3_, IncFII_pHN7A8_, IncR, IncX3, ColRNAI |
| 33 | IncFIB_(K)-1-kpn3_, IncFII_pHN7A8_, IncR, IncX3, ColRNAI |
| 34 | IncFII_pHN7A8_, IncFII_pKP91_, IncR, ColRNAI |
| 35 | IncFII_pHN7A8_, IncR, IncN, ColRNAI |
| 36 | IncFII_pHN7A8_, IncR, IncQ1, ColpVC |
| 37 | ColpVC |
| 38 | IncFII_pHN7A8_, IncR, IncN |
| 39 | IncFIA, IncFIB_(K)-1-kpn3_, IncFII_pHN7A8_, IncR, ColRNAI |
| 40 | IncR, ColpVC, repB |
| 41 | IncFIA, IncR, IncN, IncU, ColpVC |
| 42 | IncFIB_(K)-1-kpn3_, IncFII_pHN7A8_, IncR, ColRNAI, Col156, ColpVC, repB |
| 43 | IncFIB_(K)-1-kpn3_, IncFIB_AP001918_, IncFII_pHN7A8_, IncR, Col156, repB |
| 44 | IncFIB_(K)-1-kpn3_, IncFII_pHN7A8_, IncR, ColRNAI, Col156, repB |
| 45 | IncFII_pHN7A8_, IncN |
| 46 | IncFIB_(K)-1-kpn3_, IncFII_pHN7A8_, IncR, ColRNAI, Col156, ColpVC, repB |
| 47 | IncFIB_(K)-1-kpn3_, IncR |
| 48 | IncFII_pHN7A8_, IncN, IncU, ColRNAI, ColpVC |
| 49 | IncR, Col440I |
| 50 | IncFIB_(K)-1-kpn3_, IncFIB_AP001918_, IncR, IncX3 |
| 51 | IncFIB_(K)-1-kpn3_, IncFIB_AP001918_, IncR, IncX3 |
| 52 | IncFII_pHN7A8_, IncR, ColRNAI |
| 53 | IncFII_pHN7A8_, IncR, ColRNAI |
| 54 | IncFII_pHN7A8_, IncR, ColRNAI |
| 55 | IncFIA, IncFII_pHN7A8_, IncR, ColRNAI |
| 56 | IncFII_pHN7A8_, IncR, IncL, ColRNAI, Col440II |
| 57 | IncFIA, IncR |
| 58 | None |
| 59 | IncC, IncFIB_(K)-1-kpn3_, IncFIB_AP001918_, IncR, IncN |
| 60 | IncFII_pHN7A8_, IncR, ColRNAI |
| 61 | IncFIB_(K)-1-kpn3_, IncR, IncM1, IncP6 |
| 62 | IncFII_pHN7A8_, IncR, ColRNAI |
| 63 | IncFII_pHN7A8_, IncR, ColRNAI |
| 64 | IncFII_pHN7A8_, IncR, ColRNAI |
| 65 | IncI1-I |
| 66 | IncR, Col440I |
| 67 | IncFIB_AP001918_, IncFII_pHN7A8_ |
| 68 | IncFII_pHN7A8_, IncR, ColRNAI |
| 69 | IncFIB_(K)-1-kpn3_, ColRNAI |
| 70 | IncFIB_(K)-1-kpn3_, IncR, ColRNAI, Col156, repB |
| 71 | IncR, IncX3 |
| 72 | IncFII_pHN7A8_, IncR, ColRNAI |
| 73 | IncX3 |
| 74 | IncI2 |
| 75 | IncFIB_(K)-1-kpn3_, IncFII_pHN7A8_, IncR, ColRNAI, Col156, repB |
| 76 | IncFIB_(K)-1-kpn3_, IncFII_pHN7A8_, IncR, ColRNAI, Col156, repB |
| 77 | IncFIB_(K)-1-kpn3_, IncFII_pHN7A8_, IncR, ColRNAI, Col156 |
| 78 | IncFIB_(K)-1-kpn3_, IncFIB_AP001918_, IncR |
| 79 | IncFIB_(K)-1-kpn3_, IncFII_pHN7A8_, IncR, ColRNAI, Col156, repB |
| 80 | IncR, IncQ1, IncX3, repB |
| 81 | IncFII_pHN7A8_ |
| 82 | IncR, Col440I |
| 83 | IncFII_pKPX1_, IncI2 |
| 84 | IncFII_pKPX1_, IncN |
| 85 | IncFIB_(K)-1-kpn3_ |
|  |  |
| 86 | IncFIB_(K)-1-kpn3_, IncX3 |
| 87 | IncFIB_(K)-1-kpn3_, IncFIB_AP001918_, IncFII_pHN7A8_, IncR, ColRNAI |
| 88 | IncFIA, IncFII_pHN7A8_, ColRNAI |
| 89 | IncFII_pHN7A8_, IncR, ColRNAI |
| 90 | IncFII_pHN7A8_, ColRNAI |
| 91 | IncFIA, IncFIB_(K)-1-kpn3_ |
| 92 | IncFII_pHN7A8_, IncR, ColRNAI |
| 93 | IncFIB_(K)-1-kpn3_ |
| 94 | IncFIB_(K)-1-kpn3_, IncQ1, IncX3 |
| 95 | IncFIB_(K)-1-kpn3_, IncX3 |
| 96 | IncFIB_(K)-1-kpn3_, IncFII_pHN7A8_, IncR, ColRNAI |
| 97 | IncFIB_(K)-1-kpn3_, IncFII_pHN7A8_, IncR, ColRNAI |
| 98 | IncFII_pHN7A8_, IncR, IncX3, ColRNAI |
| 99 | IncFIB_(K)-1-kpn3_, IncFII_pHN7A8_, IncFII_p14_, IncR, IncX3, ColRNAI |
| 100 | IncFIB_(K)-1-kpn3_, IncFII_pHN7A8_, IncFII_p14_, IncR, IncX3, ColRNAI |
| 101 | IncFII_pHN7A8_, IncR, ColRNAI |
| 102 | IncFII_pHN7A8_, IncR, ColRNAI |
| 103 | IncFII_pHN7A8_, IncR, ColRNAI |
| 104 | IncFII_pHN7A8_, IncFII_p14_, IncR, ColRNAI, Col156, repB |
| 105 | IncFIB_(K)-1-kpn3_, IncFII_pHN7A8_, IncR, ColRNAI |
| 106 | IncFIA, IncFII_pHN7A8_, IncR, ColRNAI |
| 107 | IncFII_p14_, IncR, IncX3, ColRNAI |
| 108 | IncFIB_(K)-1-kpn3_ |
| 109 | IncR, ColRNAI |
| 110 | IncFIA, IncFII_pHN7A8_, IncR, IncN, ColRNAI |
| 111 | IncFIB_(K)-1-kpn3_ |
| 112 | IncR, ColRNAI |
| 113 | IncFII_pHN7A8_, IncR, ColRNAI |
| 114 | IncFII_pHN7A8_, IncR, ColRNAI |
| 115 | IncFII_pHN7A8_, IncR, ColRNAI |
| 116 | IncFIB_(K)-1-kpn3_, IncI1-I |
| 117 | IncFIB_(K)-1-kpn3_, IncX3 |
| 118 | IncFIB_(K)-1-kpn3_, IncFII_pHN7A8_, IncR, ColRNAI |
| 119 | IncFII_pHN7A8_, IncR, IncX3, ColRNAI |
| 120 | IncFIB_(K)-1-kpn3_, IncFII_pHN7A8_, IncR, IncX3, ColRNAI, Col440I |
| 121 | IncFIB_(K)-1-kpn3_, IncFII_pHN7A8_, IncR, IncX3, ColRNAI |
| 122 | IncFIB_(K)-1-kpn3_, IncFII_pHN7A8_, IncR, IncX3, ColRNAI |
| 123 | IncFIB_(K)-1-kpn3_, IncFII_pHN7A8_, IncR, IncX3, ColRNAI |
| 124 | IncFIB_(K)-1-kpn3_, IncFII_pHN7A8_, IncR, IncX3, ColRNAI |
| 125 | IncFIB_(K)-1-kpn3_, IncFII_pHN7A8_, IncR, ColRNAI, Col156, repB |
| 126 | IncFIB_(K)-1-kpn3_, IncFII_pHN7A8_, IncR, IncX3, ColRNAI |
| 127 | IncFIB_(K)-1-kpn3_, IncFII_pHN7A8_, IncR, ColRNAI |
| 128 | IncFII_pHN7A8_, IncR, IncX3, ColRNAI |
| 129 | None |
| 130 | IncFII_pHN7A8_, IncR, ColRNAI |
| 131 | IncFII_pHN7A8_, IncR, ColRNAI |
| 132 | IncFII_pHN7A8_, ColRNAI |
| 133 | IncFIB_(K)-1-kpn3_, IncFII_pHN7A8_, IncR, IncX3, ColRNAI |
| 134 | IncFIB_(K)-1-kpn3_, IncFII_pHN7A8_, IncR, IncX3, ColRNAI |
| 135 | IncFIB_(K)-1-kpn3_, IncFII_pHN7A8_, IncR, IncX3, ColRNAI |
| 136 | IncFIB_(K)-1-kpn3_, IncN |
| 137 | IncFIB_(K)-1-kpn3_, IncFII_pHN7A8_, IncR, IncX3, ColRNAI |
| 138 | IncR, Col440I |
| 139 | IncR, Col440I |
| 140 | Col440I |
